# Supplementary material for: Quantitative description of the phase-separation behavior of the multivalent SLP65–CIN85 complex
Source: PNAS Nexus. 2024 Feb 14;3(3):pgae079. doi: 10.1093/pnasnexus/pgae079 (PMC10923291; doi:10.1093/pnasnexus/pgae079)
Supplement: pgae079_Supplementary_Data [file PNASNEXUS-PNASNEXUS-2023-01281R-s01.pdf › PNASNEXUS-PNASNEXUS-2023-01281R-s01.pdf]

Max-Planck-Institut für multidisziplinäre Naturwissenschaften

NMR-based structural biology
Prof. Dr. Dr. h.c. C. Griesinger
Am Faßberg 11
D-37077 Göttingen
Telefon +49-(0)551-201-0
Durchwahl +49-(0)551-201-2201
Fax +49-(0)551-201-2202
Email: cigr@mpinat.mpg.de
Datum 24.11.23

Editors of PNAS Nexus

Dear Editor,

we would like to submit the manuscript entitled "Quantitative description of the phase separation behavior of the multivalent SLP65-CIN85 complex" by
Joachim Maier, Daniel Sieme, Leo E. Wong, Furqan Dar, Jürgen Wienands, Stefan Becker, and myself to PNAS Nexus.
Our work describes the phase separation of two multivalent proteins in an approach using biochemically reconstituted condensates and a computational model from the software LASSI (LAttice simulation engine for Sticker and Spacer Interactions, Choi et al. PLoS Comput Biol, 2019) by the principle of thermodynamics and a coarse-grained structural representation of sticker-spacer interactions. In short, signal transduction in B cells relies on the pre-organization of two effector proteins SLP65 and CIN85 within phase-separated condensates. The condensation is driven by the interaction of three Src homology 3 (SH3) domains of CIN85 with 6 proline-rich motifs (PRM) in SLP65. We derive from the 18 individual Kds of the interactions the thermodynamics of phase separation for wt but also designer mutants faithfully using the program LASSI. In the course of the investigation, we discovered that CIN85-SH3C did not participate in phase separation with SLP65. In parallel, we had identified a previously unknown intramolecular interaction between CIN85-SH3C and CIN85-PRM1 (Sieme et al. bioarxiv 2023) that perfectly is in line with the phase separation behavior. Altogether, our manuscript shows that LASSI can be parameterized to obtain a faithfully description of the SLP65/CIN85 condensate. Within that framework, LASSI results could be used as a heuristic tool to find previously undiscovered interactions. Readers are likely to find our manuscript interesting due to the interdisciplinary approach including a parameterized computational model and in vitro experiments.
The manuscript had been reviewed at PNAS and based on the feedback of the two reviews the editor concluded that that the manuscript was technically fine but not novel enough for PNAS. The two reviewers' comments and our replies to them including the changes to the manuscript are attached below.

With best regards,

(Prof. Dr. Dr. h.c. Christian Griesinger)

Reviewer Comments from PNAS reviewers:
Reviewer #1:

Suitable Quality?: Yes
Sufficient General Interest?: Yes
Conclusions Justified?: Yes
Clearly Written?: Yes
Procedures Described?: Yes
Supplemental Material Warranted?: Yes
Comments on Significance Statement:
LASSI has previously been used to describe the phase behavior of multivalent proteins, and the statement describing this as a new achievement should be modified.
Reply: The significance statement was modified. It reads now:

Significance Statement

Biomolecular phase separation plays an important role in cellular organization. On the molecular level, phase separation is mediated by specific amino acid sequences, as they occur in IDPs or low-complexity domains or by protein motifs/domains within multivalent proteins. The prominent SH3/PRM interaction determines the interactions between the multivalent proteins SLP65 and CIN85, the latter containing three SH3 domains. The B cell specific SLP65 contains six PRMs. We parameterized a thermodynamic computational model and describe quantitatively the phase separation originating from the underlying individual interactions of SLP65 & CIN85 using the software LASSI. LASSI faithfully revealed the determinants of phase separating these multivalent proteins.

Comments:
This manuscript by Maier et al. quantitatively characterizes the phase behavior of the multivalent proteins SLP65 and CIN85 and the interactions of their individual SH3 domains and PRM motifs. This is important downstream of B-cell receptor signaling in the immune response. Based on modeling with the coarse-grained simulation paradigm LASSI, they find that the experimental phase behavior cannot be reproduced well with the use of the experimental microscopic affinities. This discrepancy was in agreement with the identification of a PRM in the SH3-containing protein CIN85, which effectively saturates the last of the SH3 domains and therefore renders CIN85 a divalent instead of a trivalent binder. With this insight and the respective modifications to the model, the phase behavior of the two proteins can be reproduced well with the experimental microscopic affinities.

This manuscript's strengths are the large amounts of quantitative data and the clear conceptual description. The writing is somewhat dense given the large number of different protein constructs, but the authors did their best to use a clear naming strategy, and I cannot make a constructive suggestion for simplifying the text. Given the complexities of the system, they wrote a pretty clear paper. I support publication of the manuscript after the following concerns are addressed:

1. In this manuscript, the authors arrive at the conclusion that the valence of the proteins is the most important factor for determining the driving force for phase separation in a very nice way. I would argue that this is overall well known and has in fact been shown by the seminal paper from Mike Rosen's lab in 2012 (Li et al Nature 2012), and this should be clearly stated.
Reply:

We cite this paper (ref. 24) in the results and conclusion section with the following sentences:

“This finding is in line with earlier studies, which revealed that phase separation of multivalent proteins is primarily governed by valency [24, 32-34], while further determinants driving phase separations such affinity [35] and linker length [36, 37] are secondary.”
Conclusion: “These findings highlight the impact of valency on the critical concentration, as was described before in pioneering studies by Li *et al. [24]*.”

Comment: 2. LASSI has not only been used for modeling the phase behavior of IDRs. It is a package that can capture the phase behavior of any sticker/spacer system and has in fact also been used for poly-SH3/poly-PRM-type systems (see Harmon et al. eLife 2017, which the authors indeed cite as Ref 36), and e.g., for NPM1, RNA and FIB1 (Feric et al. Cell 2015).
Reply: We include in the conclusion the following sentence making reference to previous applications of LASSI:
“LASSI can be used to describe the phase separation properties of any sticker-spacer system. In biological systems, it has been successfully applied to IDPs [33], poly-SH3/poly-PRM-type [36] and multiphase systems [38].”

Comment: 3. The authors are not measuring critical concentrations but rather saturation concentrations of one of the proteins while the other one is kept constant.
Reply:
With due respect we disagree. In our study, the critical concentration is measured by varying the concentration of both proteins, which are present at a 1:1 ratio. In our experiments with the poly-SH3/poly-PRM system, the molecules are not saturated, at the very most saturation of individual PRMs or SH3 may occur.

Comment: 4. The authors describe condensates as "clusters" in several instances. While this may be a molecularly valid name, I would like to draw their attention to the fact that there is precedence for calling the higher-order complexes formed by multivalent systems below the saturation concentration "clusters" or "pre-percolation clusters". It may be useful to steer away from this name for condensates.
Reply: We replaced to term “cluster” by condensate in the appropriate context.

Reviewer #2:

Suitable Quality?: No
Sufficient General Interest?: No
Conclusions Justified?: No
Clearly Written?: Yes
Procedures Described?: Yes
Supplemental Material Warranted?: Yes

Comments:
In this manuscript, Maier et al. dissected the phase separation properties of the SLP65/CIN85 complex system using its natural and designed forms of proteins. The SLP65/CIN85 complex is a SH3/PRM-based multivalent system that is popular as a model system for investigating mechanisms of biomolecular phase separation. The authors characterized the contributions of each SH3 in CIN85 and PRMs in SLP65 in the phase separation of the SLP65/CIN85 and used the LASSI model to simulate the phase separation behavior of the natural and designed forms of the complexes. It appears that the LASSI model can predict the phase separation threshold concentration of the SLP65/CIN85 system mixed at an equal molar ratio.

Though this work provides a detailed molecular picture underlying the contributions of each molecular elements (i.e., different SH3 domains and various PRMs) to the phase separation of the SLP65/CIN85 complex, it falls short by not being able to generate new insights into general principles for understanding biomolecular phase separation. In addition, the results of the work do not seem to provide too much additional new knowledge for understanding BCR signaling. Thus, this work does not seem to be a strong candidate for PNAS.

Other minor concerns:

1. The error bars of the dispersion index profiles are too large (e.g., Fig. 3B-D) to calculate the precise critical concentrations in the phase separation assays.

Reply: The precision depends on the sampling and the range of the investigated concentration range. In the dispersion index profiles, there are simulations with either all simulations in a dispersed state or a phase separated state. We take these borders into account to report the error of the simulated critical concentrations (shown in Fig. 4).

Comment: 2. The quality of the fluorescence images of the SLP65/CIN85 condensates is not high for some figures (e.g., Fig. 2B, Fig. S7), making it hard to judge the qualities of the figures.

Reply: We replaced the low resolution images of Figure 2B and Figures S7 and S8 with high resolution ones.
